# Simultaneous hydrogen and ethanol production from cascade utilization of mono-substrate in integrated dark and photo-fermentative reactor

**DOI:** 10.1186/s13068-014-0191-x

**Published:** 2015-01-22

**Authors:** Bing-Feng Liu, Guo-Jun Xie, Rui-Qing Wang, De-Feng Xing, Jie Ding, Xu Zhou, Hong-Yu Ren, Chao Ma, Nan-Qi Ren

**Affiliations:** State Key Laboratory of Urban Water Resource and Environment, Harbin Institute of Technology, Harbin, 150090 China; Advanced Water Management Centre, The University of Queensland, St. Lucia, QLD 4072 Australia

**Keywords:** Hydrogen production, Ethanol production, Dark-fermentation, Photo-fermentation, Integrated dark and photo-fermentative reactor, Kinetics, Membrane

## Abstract

**Background:**

Integrating hydrogen-producing bacteria with complementary capabilities, dark-fermentative bacteria (DFB) and photo-fermentative bacteria (PFB), is a promising way to completely recover bioenergy from waste biomass. However, the current coupled models always suffer from complicated pretreatment of the effluent from dark-fermentation or imbalance between dark and photo-fermentation, respectively. In this work, an integrated dark and photo-fermentative reactor (IDPFR) was developed to completely convert an organic substrate into bioenergy.

**Results:**

In the IDPFR, *Ethanoligenens harbinese* B49 and *Rhodopseudomonas faecalis* RLD-53 were separated by a membrane into dark and photo chambers, while the acetate produced by *E. harbinese* B49 in the dark chamber could freely pass through the membrane into the photo chamber and serve as a carbon source for *R. faecalis* RLD-53. The hydrogen yield increased with increasing working volume of the photo chamber, and reached 3.38 mol H_2_/mol glucose at the dark-to-photo chamber ratio of 1:4. Hydrogen production by the IDPFR was also significantly affected by phosphate buffer concentration, glucose concentration, and ratio of dark-photo bacteria. The maximum hydrogen yield (4.96 mol H_2_/mol glucose) was obtained at a phosphate buffer concentration of 20 mmol/L, a glucose concentration of 8 g/L, and a ratio of dark to photo bacteria of 1:20. As the glucose and acetate were used up by *E. harbinese* B49 and *R. faecalis* RLD-53, ethanol produced by *E. harbinese* B49 was the sole end-product in the effluent from the IDPFR, and the ethanol concentration was 36.53 mmol/L with an ethanol yield of 0.82 mol ethanol/mol glucose.

**Conclusions:**

The results indicated that the IDPFR not only circumvented complex pretreatments on the effluent in the two-stage process, but also overcame the imbalance of growth and metabolic rate between DFB and PFB in the co-culture process, and effectively enhanced cooperation between *E. harbinense* B49 and *R. faecalis* RLD-53. Moreover, simultaneous hydrogen and ethanol production were achieved by coupling *E. harbinese* B49 and *R. faecalis* RLD-53 in the IDPFR. According to stoichiometry, the hydrogen and ethanol production efficiencies were 82.67% and 82.19%, respectively. Therefore, IDPFR was an effective strategy for coupling DFB and PFB to fulfill efficient energy recovery from waste biomass.

## Background

Energy shortages and environmental pollution resulting from fossil fuels have been gaining global concern and driving worldwide use of renewable energy to achieve a more secure, reliable, and sustainable energy system [1,2]. Hydrogen has been attracting great interest as a clean, renewable, and effective energy carrier that could minimize our dependence on fossil fuel-derived energy and therefore enhance the global economy and reduce environmental pollution. Nevertheless, nearly 96% of hydrogen is produced through thermochemical processes using fossil fuel as an energy source [3], which is energy intensive, unsustainable, and not environmentally friendly. In contrast, microbial hydrogen recovery from renewable sources like organic wastes and sunlight is mostly operated at ambient temperatures and pressures [4]. Therefore, biological hydrogen production is considered an important step to a sustainable world power supply with the potential to replace fossil fuels [5].

Photo and dark-fermentation are the main pathways for biological hydrogen production. Photo-fermentative bacteria (PFB) could convert 100% of organic wastes into hydrogen and carbon dioxide by harvesting energy from sunlight. However, most PFB live on short-chain fatty acids, such as acetic, propionic, and butyric acids [6-8] and can hardly use glucose and other macromolecular organics. This greatly limits hydrogen production from complex organic wastes through photo-fermentation. Conversely, dark-fermentative bacteria (DFB) produce hydrogen from various complex organic wastes at high rates via butyric acid-type, propionic acid-type, or ethanol-type fermentation [9], but the hydrogen yield is limited by end products such as acetic, propionic, and butyric acids. As a result, the maximum hydrogen yield for dark-fermentation is 4 mol H_2_/mol hexose, which is far from the theoretical maximum value of 12 mol H_2_/mol hexose [5,10]. However, the short-chain fatty acids produced by DFB provide a carbon source for cell growth and electron donors for hydrogen production by PFB. Therefore, combining DFB and PFB with complementary capabilities is a promising way to completely convert complex organic wastes into hydrogen.

Currently, the combination of dark and photo-fermentation is mainly achieved through a two-stage or co-culture process. In the two-stage process, the effluent from dark-fermentation serves as the carbon source for photo-fermentation [11]. High hydrogen yields can be obtained by the two-stage process, because DFB and PFB work separately under their respective optimal conditions. However, the effluent from dark-fermentation contains DFB, high concentration of short-chain fatty acids leading to low pH values, and sometimes ammonium, which significantly inhibits the nitrogenase activity of PFB. As a result, the effluents have to be centrifuged to remove DFB and any colloidal materials that may interfere with light penetration [12], their pH must be adjusted by additional chemical agents, then they must be diluted with distilled water, the ammonium removed [13-17], and they may even have to be re-flushed with argon and re-sterilized before reaching the optimum conditions for photo-fermentation. These complex pretreatments on the effluent from dark-fermentation not only greatly increase the operation costs, but also make it hard for continuous operation, especially in large-scale applications.

In the co-culture process, DFB and PFB are mixed and cultured in one system. Short-chain fatty acids produced by DFB are converted *in situ* into hydrogen by PFB without complex pretreatments. This not only alleviates the end-product inhibition on dark fermentation [18], but also prevents the pH drop resulting from short-chain fatty acid accumulation. However, cell growth rates of DFB are 0.15-1.12 h^−1^ [19-21], while cell growth rates of PFB are only 0.025-0.074 h^−1^ [22-25]. DFB will eventually dominate the co-culture system, in spite of increased inoculation with PFB. Moreover, the imbalance of organic acid production and consumption rates between DFB and PFB could lead to accumulation of organic acids and decrease of pH. Compared with DFB, PFB are more sensitive to pH change; their optimum pH is from 6.5 to 7.5. A large amount of phosphates are required in the co-culture system to provide sufficient buffer capacity, which greatly increases the operation costs. In addition, PFB suffer a light shading effect from DFB in the co-culture system, which could decrease the light conversion efficiency. As a result, it is difficult to set up a stable co-culture process.

The objective of this study was to develop a novel hydrogen production mode by coupling dark and photo-fermentation to take full advantage of the complementary capabilities of DFB and PFB and reduce operating costs. The kinetic characteristics of growth and hydrogen production of the DFB, *Ethanoligenens harbinese* B49, and the PFB, *Rhodopseudomonas faecalis* RLD-53, were determined. Based on the kinetics of DFB and PFB, we designed a novel integrated dark and photo-fermentative reactor (IDPFR). The hydrogen production of this novel reactor was also investigated under different operating conditions.

## Results and discussion

### Kinetics of dark and photo-fermentative bacteria

**Figure 1:**
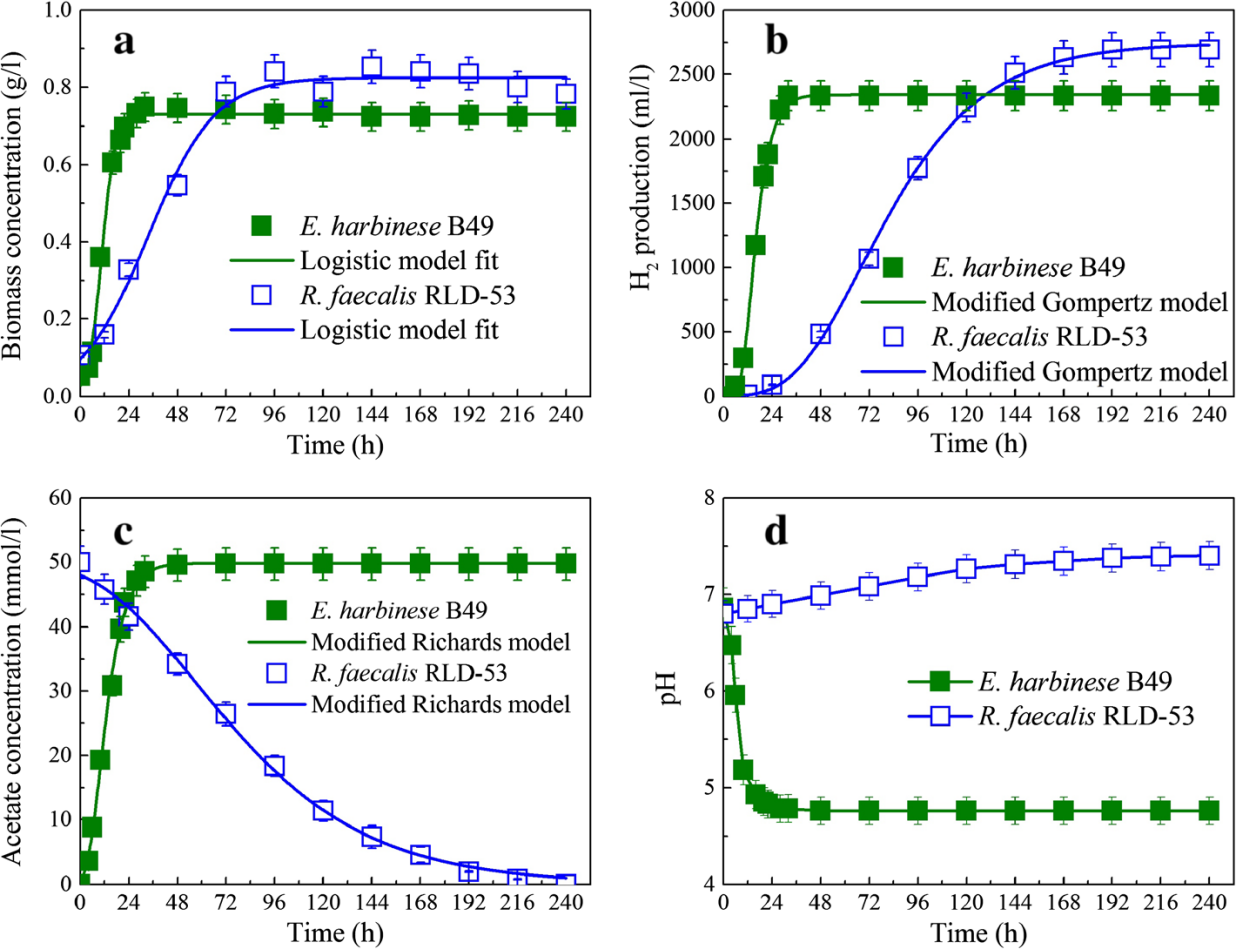
**Kinetic characterizations of dark and photo-fermentative bacteria. (a)** Cell growth kinetics; **(b)** H_2_ production kinetics; **(c)** acetate production and consumption kinetics; **(d)** pH change during fermentation.

The kinetics of cell growth, hydrogen production, acetic acid production, and consumption of *E. harbinese* B49 and *R. faecalis* RLD-53 were investigated under their own optimal conditions [26,27] with a logistic model, modified Gompertz equation, and modified Richards model, respectively (Figure 1), and the main kinetic parameters are summarized in Table 1. At a glucose concentration of 10 g/L, the maximum hydrogen production rate (${R}_{H_2}$) of *E. harbinense* B49 was 163.98 ml/L/h, which was more than five times that of *R. faecalis* RLD-53. 49.84 mmol/L of acetate was produced by *E. harbinense* B49 with a maximum production rate (*R*_*pHAc*_) of 2.73 mmol/L/h, while the maximum acetate degradation rate (*R*_*dHAc*_) by *R. faecalis* RLD-53 was only 0.38 mmol/L/h at an acetate concentration of 50 mmol/L (Table 1). The results showed that the maximum acetate production rate by *E. harbinense* B49 was about seven times the degradation rate by *R. faecalis* RLD-53. In addition, the specific growth rate (*k*_*c*_) of *E. harbinense* B49 was 0.31 h^−1^, which indicated that *E. harbinense* B49 grows more slowly than *Clostridium butyricum* CGS5 with a specific growth rate of 0.77 h^−1^ [20] and *Enterobacter cloacae* IIT-BT 08 with a specific growth rate of 1.12 h^−1^ [21]. The specific growth rate (*k*_*c*_) of *R. faecalis* RLD-53 was 0.06 h^−1^, which was much faster than *Rhodobacter capsulatus* DSM 1710 with a specific growth rate of 0.025 h^−1^ [22], but slightly slower than *Rhodopseudomonas palustris* with a specific growth rate of 0.074 h^−1^ [24]. The results also showed that the specific growth rate of *E. harbinense* B49 was about five times faster than that of *R. faecalis* RLD-53 (Table 1). Therefore, the imbalance of metabolic and cell growth rate between the two types of bacteria could exacerbate the accumulation of acetic acid, which would decrease the pH and subsequently inhibit *R. faecalis* RLD-53. In the IDPFR, a membrane with pore size 0.22 μm was used to divide the reactor into two separate reaction chambers. In the dark chamber, complex waste biomass was converted by *E. harbinense* B49 into hydrogen, carbon dioxide, ethanol, and acetate. Acetate could diffuse through the membrane into the photo chamber. In the photo chamber, acetate from the dark chamber was utilized by *R. faecalis* RLD-53 to produce hydrogen and carbon dioxide. By increasing the working volume of the photo chamber, more *R. faecalis* RLD-53 could couple with *E. harbinense* B49, and the organic acids produced by *E. harbinense* B49 could be expected to be consumed completely by *R. faecalis* RLD-53 without accumulation.

**Table 1:** **The kinetic parameters of dark and photo-fermentative bacteria**

**Kinetic parameters**	***E. harbinense*** **B49**	***R. faecalis*** **RLD-53**
*X*_max_ (g/L)	0.73	0.82
*k*_*c*_ (h^−1^)	0.31	0.06
*H*_max_ (ml/L)	2342.18	2737.45
${R}_{H_2}$ (ml/L/h)	163.98	30.47
${\lambda}_{H_2}$ (h)	8.66	35.54
*R*_*pHAc*_ and *R*_*dHAc*_ (mmol/L/h)	2.73	0.39
pH	6.80-4.75	6.80-7.42

### H_2_ production by IDPFR at different working volume ratios

In the IDPFR, the working volume ratio of the dark and photo chambers was investigated to overcome the imbalance of cell growth and metabolic rate between *E. harbinense* B49 and *R. faecalis* RLD-53. The working volume of the dark chamber was a constant value of 40 ml, while the working volume of the photo chamber was 80, 120, 160, and 200 ml, corresponding to a working volume ratio at 1:2, 1:3, 1:4, and 1:5. In this test, the phosphate buffer concentration was 10 mmol/L, and the initial glucose concentration was 10 g/L.

**Figure 2:**
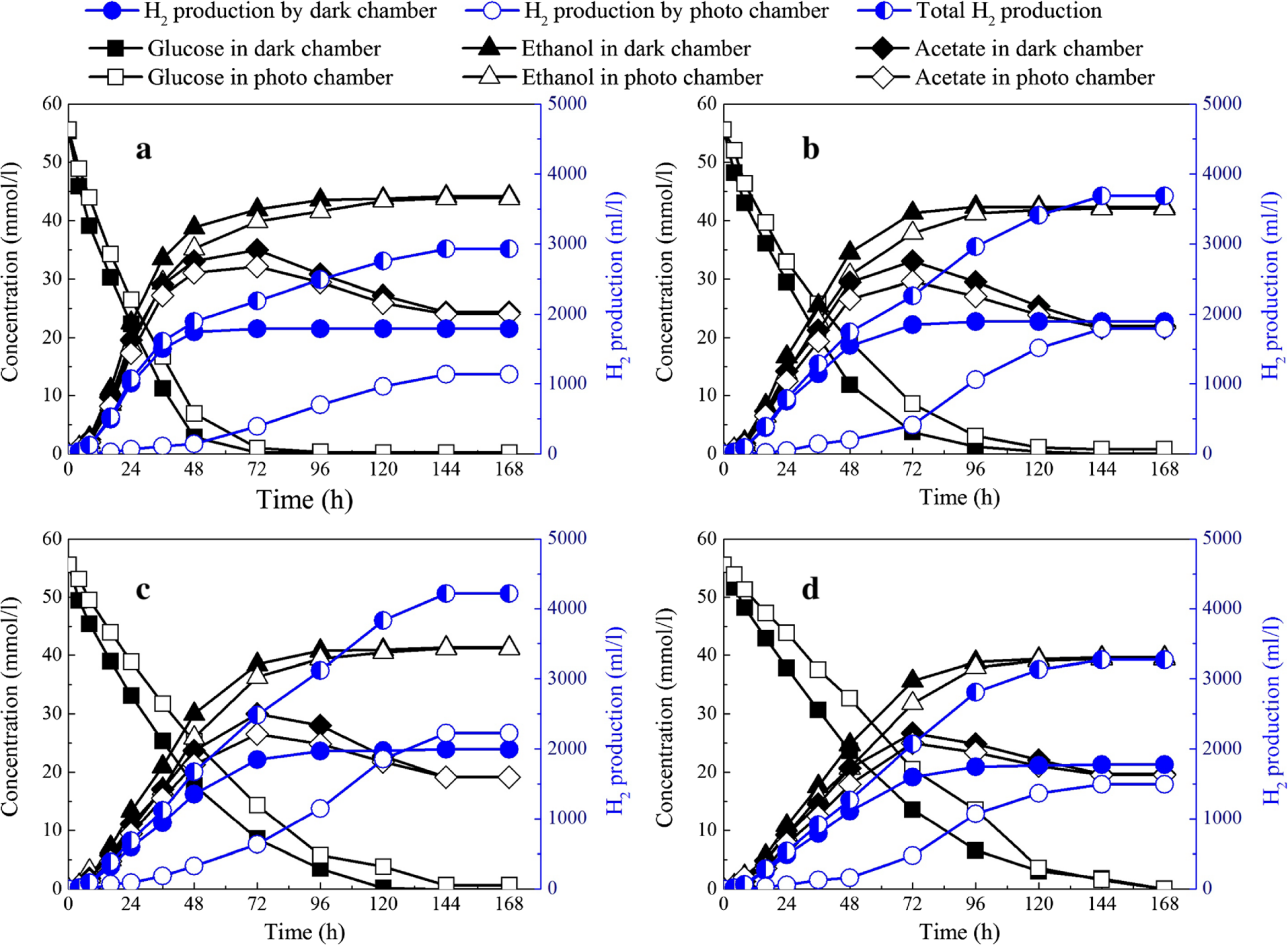
**Hydrogen production, glucose consumption, and acetate accumulation in IDPFR at different working volume ratios of dark chamber to photo chamber. (a)** 1:2; **(b)** 1:3; **(c)** 1:4; **(d)** 1:5.

The hydrogen production volume increased with dark-to-photo chamber ratio from 1:2 to 1:4, and decreased with a ratio from 1:4 to 1:5 (Figure 2). At a working volume ratio of 1:2, hydrogen production by the dark chamber was 1,791.67 ml/L, while hydrogen production by the photo chamber was only 1,138.97 ml/L. The total hydrogen production was 2,930.63 ml/L with a hydrogen yield of 2.35 mol H_2_/mol glucose. With an increase of photo chamber working volume, more *R. faecalis* RLD-53 could use the end product from *E. harbinense* B49 for hydrogen production, while *E. harbinense* B49 was restricted in the small dark chamber. Acetic acid produced by *E. harbinense* B49 was not accumulated but converted into hydrogen by *R. faecalis* RLD-53. Consequently, hydrogen production by photo chamber increased significantly. At a working volume ratio of 1:4, hydrogen production by the dark and photo chambers reached maximum simultaneously at 1,991.67 and 2,225.15 ml/L, with a hydrogen yield of 3.38 mol H_2_/mol glucose. However, with a further decrease of the working volume ratio to 1:5, total hydrogen production decreased sharply to 3,269.29 ml/L with a hydrogen yield of 2.63 mol H_2_/mol glucose.

The glucose concentration in the dark chamber gradually decreased due to consumption by *E. harbinense* B49. The glucose diffused spontaneously from the photo to the dark chamber for further consumption by *E. harbinense* B49. Consequently, the glucose concentration in the photo chamber was slightly higher than that in the dark chamber, but glucose concentration in both chambers decreased following the same trend. Acetate diffused into the photo chamber through the membrane and was converted by RLD-53 into hydrogen and carbon dioxide. The acetate production rate by B49 was much faster than the consumption rate of *R. faecalis* RLD-53. Consequently, acetate accumulated in the reactor, and reached maximum value at about 72 h. The maximum accumulation of acetate decreased from 34.95 mmol/L to 26.72 mmol/L with the increase of photo chamber working volume. A previous study showed that acetate has been proved to have an inhibitory effect on hydrogen production by *E. harbinese* B49 [28]. In the IDPFR, acetate consumed by *R. faecalis* RLD-53 could alleviate the inhibition of acetate on hydrogen production and also facilitate a stable pH of the coupled system.

### Effect of phosphate concentration on H_2_ production by IDPFR

In the IDPFR, phosphate not only serves as a phosphorus source for microbial growth, but also can buffer the pH change in the fermentation system. Hydrogen production by the IDPFR was investigated at different phosphate concentrations (10, 20, and 30 mmol/L). In this test, the working volume of the dark chamber was 40 ml, while the working volume of the photo chamber was 160 ml, corresponding to a total working volume of 200 ml.

**Figure 3:**
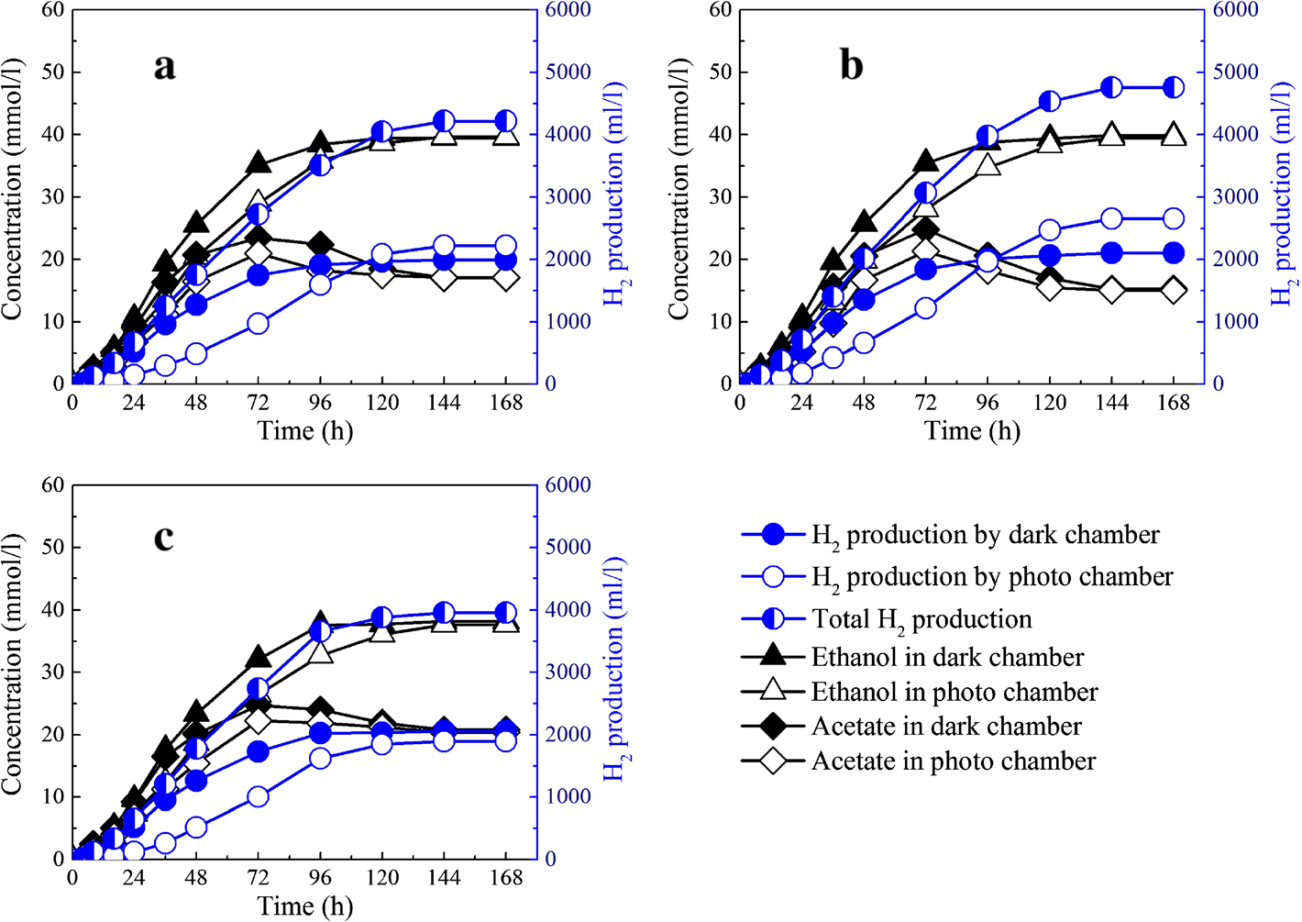
**Hydrogen production and acetate accumulation in IDPFR at different phosphate concentrations. (a)** 10 mmol/L; **(b)** 20 mmol/L; **(c)**, 30 mmol/L.

As shown in Figure 3, at a phosphate concentration of 10 mmol/L, the total hydrogen production was 4,199.14 ml/L and the hydrogen yield reached 3.37 mol H_2_/mol glucose. When the phosphate concentration increased to 20 mmol/L, the total hydrogen production was 4,766.90 ml/L and the hydrogen yield reached 3.83 mol H_2_/mol glucose. The result indicated that the increase of phosphate concentration from 10 to 20 mmol/L was conducive to hydrogen production by dark and photo-fermentation. However, with a further increase of phosphate concentration to 30 mmol/L, hydrogen production by *R. faecalis* RLD-53 in the photo chamber decreased sharply to 1,893.93 ml/L, and the hydrogen yield was only 3.18 mol H_2_/mol glucose. The high concentration of phosphate also inhibited hydrogen production by *R. faecalis* RLD-53, and therefore decreased hydrogen production by the IDPFR. With an increase of phosphate concentration from 10 mmol/L to 20 mmol/L, acetate concentration in the end product decreased from 17.03 to 13.04 mmol/L. Consequently, the end pH of the IDPFR increased from 5.8 to 6.2, which approximated the optimum pH (6.5-7.5) for *R. faecalis* RLD-53. However, when the phosphate concentration was further increased to 30 mmol/L, the acetate concentration significantly increased to 20.78 mmol/L, probably due to inhibition of cell growth and hydrogen production activities resulting from the high phosphate concentration. As a result, acetate accumulation increased and therefore the end pH decreased to 5.6, which is unfavorable for PFB. Therefore, the maximum hydrogen yield was obtained at a phosphate concentration of 20 mmol/L.

In the co-culture system, the imbalance of acetate production by *E. harbinense* B49 and consumption by *R. faecalis* RLD-53 and the pH-sensitive PFB necessitate a high buffer capacity of the coupled system by adding phosphate. Hydrogen production by co-culture of *E. harbinense* B49 and immobilized *R. faecalis* RLD-53 reached a maximum 2.78 mol H_2_/mol glucose at a phosphate concentration of 50 mmol/L [29]. However, a life cycle inventory analysis of biological hydrogen production by coupling dark and photo-fermentation showed that 53.5% of the environmental impact is generated by the use of phosphate in the fermentation processes [30]. Compared with the co-culture of *E. harbinense* B49 and *R. faecalis* RLD-53 [29], the IDPFR could significantly decrease the usage of phosphate and therefore reduce the environmental impact.

### Effect of glucose concentration on H_2_ production by IDPFR

In the coupled system, glucose concentration not only directly affects cell growth and hydrogen production of DFB, but also influences pH stability through intermediate products (such as acetate and butyrate) accumulation, which is crucial for hydrogen production and cell growth of PFB. Hydrogen production by the IDPFR was investigated at different glucose concentrations (4, 8, and 12 g/L). In this test, the working volume ratio of dark to photo chamber was 1:4, and the phosphate concentration was 20 mmol/L.

**Figure 4:**
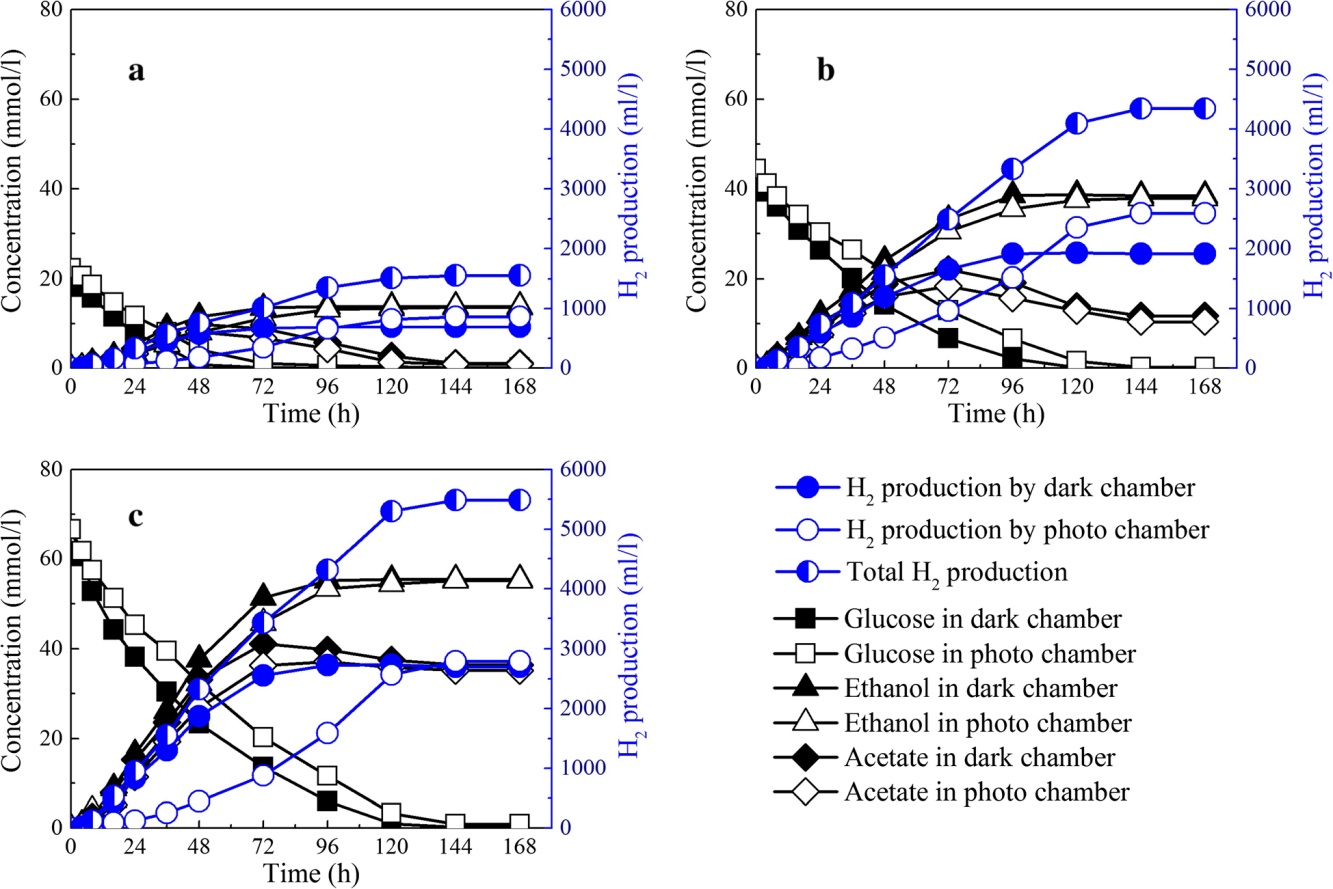
**Hydrogen production, glucose consumption, and acetate accumulation in IDPFR at different initial glucose concentrations. (a)** 4 g/L; **(b)** 8 g/L; **(c)** 12 g/L.

As shown in Figure 4, at glucose concentration of 4 g/L, the hydrogen production by the dark and photo chambers was 690.05 ml/L and 865.07 ml/L, respectively. However, glucose was exhausted at 48 h. Also, the maximum acetate accumulation was 9.93 mmol/L, which was used up at 120 h. At the low substrate concentration, most of the substrate was used for cell growth and to maintain cellular activities rather than for hydrogen production. With an increase of glucose concentration from 4 to 8 g/L, hydrogen production by the dark chamber was 1,916.92 ml/L, and hydrogen production by the photo chamber was 2,426 ml/L. As a result, the total hydrogen production reached 4,342.92 ml/L with a hydrogen yield 4.36 mol H_2_/mol glucose. However, with a further increase of glucose concentration to 12 g/L, hydrogen production by the dark chamber was further increased to 2,760.68 ml/L, but acetate rapidly accumulated in the IDPFR and the maximum concentration reached 41.13 mmol/L, due to the high glucose concentration. Excessive accumulation of acetate resulted in a rapid drop of pH, so hydrogen production of *R. faecalis* RLD-53 was severely inhibited and decreased sharply to 1,842.30 ml/L. In the co-culture of *E. harbinense* B49 and immobilized *R. faecalis* RLD-53, hydrogen production reached a maximum at a glucose concentration of 6 g/L [29], while the optimum glucose concentration was 8 g/L in the IDPFR. Therefore, the IDPFR could be operated with a low hydraulic retention time and high organic loading rate.

### Effect of bacteria inoculation ratio on H_2_ production by IDPFR

There are significant kinetic differences in cell growth and metabolic rate between dark and photo-fermentative bacteria, so the inoculation ratio of DFB to PFB may be an effective strategy for stable operation of the IDPFR. The inoculation of the dark chamber was a constant value of 1.6 mg *E. harbinense* B49, while the inoculation of the photo chamber was 16, 32, and 48 mg, corresponding to inoculation ratios of dark to photo chamber of 1:10, 1:20, and 1:30. In this test, the working volume ratio of dark to photo chamber was 1:4, the phosphate concentration was 20 mmol/L, and the glucose concentration was 8 g/L.

**Figure 5:**
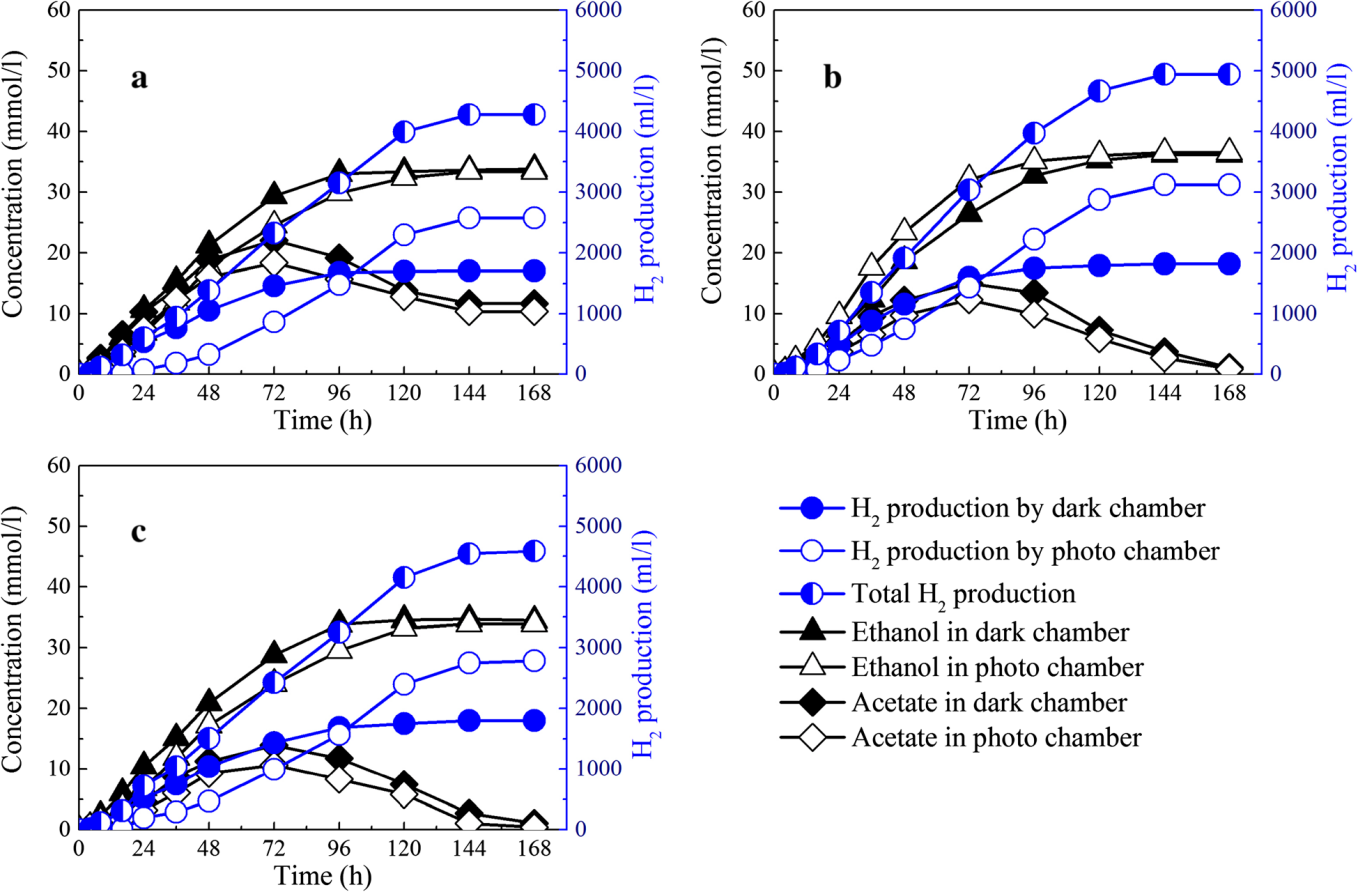
**Hydrogen production and acetate accumulation in IDPFR at different inoculation ratios of** ***E. harbinese*** **B49 to** ***R. faecalis*** **RLD-53. (a)** 1:10; **(b)** 1:20; **(c)** 1:30.

Hydrogen production by the dark and photo chambers increased with the inoculation ratio and reached a maximum 4.96 mol H_2_/mol glucose at an inoculation ratio of 1:20 (Figure 5). At an inoculation ratio of 1:20, hydrogen production by the dark chamber was 1,818.85 ml/L, while hydrogen production by the photo chamber was 3,124.22 ml/L, accounting for around 60% of the total hydrogen production. However, hydrogen production by the photo chamber decreased significantly to 2,786.30 ml/L at an inoculation ratio of 1:30, because excessive inoculation caused cells to grow quickly with less nutrition for product metabolism [31].

At an inoculation ratio of 1:20, acetate produced by *E. harbinense* B49 was effectively used by *R. faecalis* RLD-53 without excessive accumulation, so a stable match between *E. harbinense* B49 and *R. faecalis* RLD-53 was achieved and acetate was used up. Consequently, ethanol produced by *E. harbinense* B49 was the sole end product in the liquid phase with a concentration of 36.54 mmol/L, corresponding to an ethanol yield of 0.82 mol ethanol/mol glucose. According to stoichiometry, 6 mol H_2_ and 1 mol ethanol could be produced per mol glucose consumed in the IDPFR, so the hydrogen and ethanol production efficiencies achieved 82.67% and 82.19%, respectively.

## Conclusions

In this work, an integrated dark and photo-fermentative bioreactor (IDPFR) was developed based on the kinetics of dark and photo-fermentative bacteria for complete energy recovery from organic waste. In the IDPFR, the dark and photo-fermentative bacteria were separated by a membrane into dark and photo chambers, while the organic acids produced by the DFB in the dark chamber freely passed through the membrane into the photo chamber, serving as a carbon source for photo-fermentation. Increasing the working volume of the photo chamber enabled more *R. faecalis* RLD-53 to cooperate with *E. harbinense* B49, and the maximum hydrogen yield reached 3.38 mol H_2_/mol glucose at a working volume ratio of dark chamber to photo chamber of 1:4. Control of the proper phosphate buffer concentration (20 mmol/L) not only enhanced hydrogen production by maintaining a stable pH of the IDPFR, but also decreased the environmental impact caused by phosphate in the fermentation process. The maximum hydrogen production (4.96 mol H_2_/mol glucose) plus ethanol production (0.82 mol H_2_/mol glucose) were obtained by the IDPFR at a phosphate buffer concentration of 20 mmol/L, a glucose concentration of 8 g/L, and a ratio of dark-photo bacteria of 1:20. This novel reactor not only bypasses complex pretreatments on the effluent in the two-stage process, but also overcomes the imbalance of growth and metabolic rate between DFB and PFB in the co-culture process. Therefore, the IDPFR offers great advantages for enhancing production yield and scale-up application in bioenergy recovery from waste biomass.

## Material and methods

### Bacteria and media

*Ethanoligenens harbinese* B49, a typical strain used for ethanol-type fermentation, was used as the dark-fermentative bacterium. It was isolated from anaerobic activated sludge in a continuous stirred-tank reactor with ethanol-type fermentation [26]. *Rhodopseudomonas faecalis* RLD-53 was used as the photo-fermentative bacterium; it was isolated previously from freshwater pond sludge and was known to have an excellent ability for hydrogen production [27]. Acetic acid, one of the major metabolites from *E. harbinese* B49, could be further converted into hydrogen by *R. faecalis* RLD-53. The media for *E. harbinese* B49 and *R. faecalis* RLD-53 were the same as reported earlier in [26] and [27], respectively.

The medium for hydrogen production by coupling *E. harbinese* B49 and *R. faecalis* RLD-53 consisted of (in g/L) glucose, 10; sodium glutamate, 1.0; yeast extract, 2; KH_2_PO_4_, 1.36; K_2_HPO_4_, 1.74; MgCl_2_.6H_2_O, 0.2; CaCl_2_, 0.1; FeSO_4_.7H_2_O, 0.012; NaCl, 0.1; EDTA-Na_2_, 0.1; L-cysteine, 1; trace element solution, 1 ml; vitamin solution, 1 ml. The pH of the medium was adjusted to 7.0 ± 0.2 by using HCl or NaOH solutions.

### Kinetic characterizations of dark and photo-fermentative bacteria

The cell growth kinetics of *E. harbinese* B49 and *R. faecalis* RLD-53 were determined by a logistic model (Eq. 1), which has been widely used to interpret growth characteristics of hydrogen-producing bacteria [23,32]:

1 $$ x=\frac{X_{\max }}{1+\left(\frac{X_{\max }}{X_0}-1\right){e}^{-{k}_ct}} $$

where *x* is the cell concentration (g/L); *X*_*0*_ is the initial cell concentration (g/L); *k*_*c*_ is the apparent specific growth rate (h^−1^); and *X*_*max*_ is the maximum cell concentration (g/L).

The hydrogen production kinetics was examined by a modified Gompertz equation, which has been widely accepted and used to describe the cumulative hydrogen production progress [33,34]:

2 $$ H={H}_{\max } \exp \left\{- \exp \left[\frac{R_{H_2}e}{H_{\max }}\left({\lambda}_{H_2}-t\right)+1\right]\right\} $$

where *t* denotes culture time (h); *H* denotes cumulative hydrogen production (ml H_2_/L medium); *H*_*max*_ denotes maximum cumulative hydrogen production (ml H_2_/L medium); e = 2.71828; ${R}_{H_2}$ denotes maximum H_2_ production rate (ml/L/h); and ${\lambda}_{H_2}$ denotes the lag-phase time (h) for hydrogen production.

In order to make the kinetics of production and degradation of acetic acid comparable, the modified Richards model was used to describe the product formation and substrate degradation simultaneously. To describe the product formation, the Richards function was [35]:

3 $$ P={K}_p{\left\{1+\left(m-1\right){e}^m \exp \left[\frac{R_{pHAc}}{K_p}{m}^{m/\left(m-1\right)}\left({\lambda}_{pHAc}-t\right)\right]\right\}}^{1/\left(1-m\right)} $$

where *m* denotes shape parameter; *t* denotes culture time (h); *P* denotes cumulative product concentration (mmol/L medium); *K*_*p*_ denotes the product formation potential (mmol/L medium); *R*_*HAc*_ denotes maximum acetate formation rate (mmol/L/h); and λ_*pHAc*_ denotes the lag-phase time (h) for production of acetic acid.

To describe the degradation of a substrate, the Richards function can be rewritten as [36]:

4 $$ S={S}_0\left\{1-{\left\{1+\left(m-1\right){e}^m \exp \left[\frac{R_{dHAc}}{S_0}{m}^{m/\left(m-1\right)}\left({\lambda}_{dHAc}-t\right)\right]\right\}}^{1/\left(1-m\right)}\right\} $$

where *m* denotes shape parameter; *t* denotes culture time (h); *S* denotes substrate concentration (mmol/L medium); *S*_*0*_ denotes the initial substrate concentration (mmol/L medium); *R*_*dHAc*_ denotes maximum acetate consumption rate (mmol/L/h); and λ_*dHAc*_ denotes the lag-phase time (h) for degradation of acetic acid.

### Integrated dark and photo-fermentative bioreactor setup and operation

**Figure 6:**
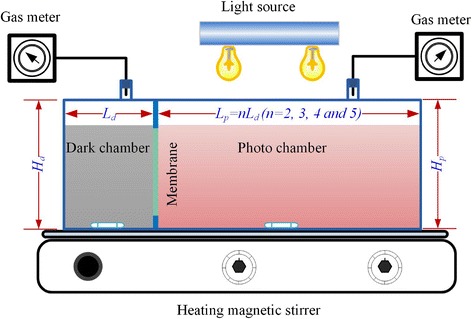
**Schematic diagram of integrated dark and photo-fermentative bioreactor.**

A schematic of the integrated dark and photo-fermentative bioreactor setup is depicted in Figure 6. The reactor was divided into dark and photo chambers by a cellulose acetate membrane with a diameter of 6 cm (pore size 0.22 μm, thickness 100 μm, Shanghai, China), which permitted the substrate to pass freely through while excluding bacterial cells. Consequently, the dark and photo-fermentative bacteria were separated into dark and photo chambers.

The illuminated area-to-working volume (A/V) ratio has a significant influence on the performance of a photobioreactor [37]. To avoid confusion between the effect of the A/V ratio and the working volume ratio of the dark and photo chambers, the IDPFR was designed to keep a constant A/V ratio when increasing the volume of the reactor. The reactor wall thickness was 10 mm. The dark chamber had the following internal dimensions: length of 30 mm (*L*_*d*_ = 30 mm), width of 50 mm (*W*_*d*_ = 50 mm), and height of 50 mm (*H*_*d*_ = 50 mm). The photo chamber was designed with the same width and height as the dark chamber, while the length of the photo chamber (*L*_*p*_) was 60, 90, 120, and 150 mm, corresponding to the working volume ratio at 1:2, 1:3, 1:4, and 1:5. As the light penetrated the reactor through the top surface of the photo chamber, the surface area receiving light energy increased with the increasing volume, reaching 30, 45, 60, and 75 cm^2^, respectively. As a result, the A/V ratio was kept constant at 37.5 m^2^/m^3^.

The IDPFRs were chemically sterilized with hydrogen peroxide (3% v/v) solution and thoroughly rinsed with distilled water. At startup, the dark and photo chambers were filled with different volumes of hydrogen production medium, and then flushed with argon gas for 10 min to maintain anaerobic conditions. *E. harbinese* B49 and *R. faecalis* RLD-53 in the mid-exponential growth phase were centrifuged and washed with a phosphate buffer solution, and inoculated into the dark and photo chambers, respectively. The liquid in the reactor was homogeneously mixed using a magnetic stirrer at 80 rpm at a constant temperature of 35 ± 1°C. The light intensity on the outside surface of the photo chambers of the reactors was maintained at 150 W/m^2^ by incandescent lamps (60 W).

### Analytical methods

Biogas was sampled from the head space of the dark and photo chambers of the reactor with gas-tight glass syringes, and the hydrogen content was determined by using a gas chromatograph (Agilent 4890D, Agilent Technologies, Santa Clara, CA, USA). The gas chromatograph column was Alltech Molesieve 5A 80/100. Argon was used as the carrier gas with a flow rate of 30 ml/min. The temperatures of the oven, injection, detector, and filament were 35, 120, 120, and 140°C, respectively. The glucose concentration in the culture broth was determined with a Glucose HK kit (Sigma). Volatile fatty acids and ethanol in the supernatant of the culture broth were determined using a second gas chromatograph (Agilent 7890 A, Agilent Technologies, USA) equipped with a flame ionization detector. The liquor samples were first centrifuged at 12,000 rpm for 5 min and then filtered through a 0.22-μm membrane before the free acids were analyzed. The operational temperatures of the injection port, the column, and the detector were 220, 190, and 220°C, respectively. Nitrogen was used as the carrier gas at a flow rate of 50 ml/min.

The light intensity was measured at the surface of the reactor with a TENMARS TM-207 Solar Power Meter (Tenmars Electronics Co., Ltd., Taiwan, China). The cell biomass was determined by filtering the culture broth through a cellulose acetate membrane filter (0.22-μm pore size, 50 mm in diameter). The filter was then rinsed with deionized water to remove salts or non-cellular materials. Each loaded filter was dried at 105°C until the weight became consistent. The dry weight of a blank filter was subtracted from that of the loaded filter to obtain the cell biomass.

## Abbreviations

λ_*dHAc*_: lag-phase time for degradation of acetic acid (h)
${\lambda}_{H_2}$: lag-phase time for hydrogen production (h)
λ_*pHAc*_: lag-phase time for production of acetic acid (h)
DFB: dark-fermentative bacteria
e: the base of the natural logarithm
*H*: cumulative hydrogen production (ml H_2_/L medium)
*H*_*max*_: maximum cumulative hydrogen production (ml H_2_/L medium)
IDPFR: integrated dark and photo-fermentative (bio)reactor
*k*_*c*_: apparent specific growth rate (h^−1^)
*K*_*p*_: product formation potential (mmol/L medium)
*m*: shape parameter
*P*: cumulative product concentration (mmol/L medium)
PFB: photo-fermentative bacteria
*R*_*dHAc*_: maximum acetate consumption rate (mmol/L/h)
${R}_{H_2}$: maximum H_2_ production rate (ml/L/h)
*R*_*pHAc*_: maximum acetate production rate (mmol/L/h)
*S*: substrate concentration (mmol/L medium)
*S*_*0*_: initial substrate concentration (mmol/L medium)
*t*: culture time (h)
*x*: cell concentration (g/L)
*X*_*0*_: initial cell concentration (g/L)
*X*_*max*_: maximum cell concentration (g/L)
